# Single-Step Protocol for Isolating the Recombinant Extracellular Domain of the Luteinizing Hormone Receptor from the *Ovis aries* Testis

**DOI:** 10.3390/cimb44110387

**Published:** 2022-11-17

**Authors:** José Luis Villalpando-Aguilar, Itzel López-Rosas, Arnulfo Montero-Pardo, Elisa Azuara-Liceaga, Javier de Jesús Valencia-Méndez, Cynthia R. Trejo-Muñoz, Carlos Kubli-Garfias

**Affiliations:** 1 Instituto de Investigaciones en Matemáticas Aplicadas y en Sistemas, Universidad Nacional Autónoma de México, Mérida 97302, Mexico; 2 Consejo Nacional de Ciencia y Tecnología, Ciudad de México 03940, Mexico; 3 Colegio de Postgraduados Campus Campeche, Campeche 24050, Mexico; 4 Facultad de Medicina Veterinaria y Zootecnia, Universidad Autónoma de Sinaloa, San Benito 80260, Mexico; 5 Posgrado en Ciencias Genómicas, Campus del Valle, Universidad Autónoma de la Ciudad de México, Ciudad de México 03100, Mexico; 6 Facultad de Medicina Veterinaria y Zootecnia, Universidad Nacional Autónoma de México, Ciudad de México 04510, Mexico; 7 Instituto de Investigaciones Biomédicas, Universidad Nacional Autónoma de México, Ciudad de México 04510, Mexico

**Keywords:** luteinizing hormone receptor, recombinant protein, G protein, *Ovis aries*, Leydig cells, pCOLD II

## Abstract

The luteinizing hormone receptor (LHR) is a glycoprotein member of the G protein-coupled receptors superfamily. It participates in corpus luteum formation and ovulation in females and acts in testosterone synthesis and spermatogenesis in males. In this study, we extracted RNA from sheep testicles and synthetized the cDNA to amplify the gene *lhr-bed*. This gene consists of 762 bp and encodes 273 amino acids of the extracellular domain of LHR. The *lhr-bed* was cloned into pJET1.2/blunt, then subcloned into pCOLD II, and finally, transformed in *E. coli* BL21 (*DE*3) cells. Because the induced rLHR-Bed protein was found in the insoluble fraction, we followed a modified purification protocol involving induction at 25 °C, subjection to denaturing conditions, and on-column refolding to increase solubility. We confirmed rLHR-Bed expression by means of Western blot and mass spectrometry analysis. It is currently known that the structure stem-loop 5′UTR on pCOLD II vector is stable at 15 °C. We predicted and obtained RNAfold stability at 25 °C. We successfully obtained the recombinant LHR extracellular domain, with protein yields of 0.2 mg/L, and purity levels of approximately 90%, by means of a single chromatographic purification step. The method described here may be used to obtain large quantities of rLHR-Bed in the future.

## 1. Introduction

The luteinizing hormone receptor (LHR) was first expressed from rat ovary cells [1] and was first categorized as an “unusual” member of the G protein-coupled receptor family. In fact, the LHR belongs to the G protein-coupled receptor (GPCR) superfamily and its ligand, and is a luteinizing hormone (LH), that is, a glycoprotein secreted by the anterior hypophysis. Structurally, the GPCRs belong to the family of seven-transmembrane domain receptors, which exhibit extracellular (NH2) and intracellular (COOH) termini. In the female ovary, LHR is located in luteal theca and follicular granulose cells. In the male testis, it is mostly found in the Leydig cells, which secrete testosterone. LHR is also expressed in the placenta [2].

Biologically, LHR is involved in crucial reproductive processes. In females, these include oocyte maturation, ovulation, and estradiol synthesis. In males, LHR participates in testosterone synthesis and sex differentiation, among other processes [3]. The receptor binds its ligand LH through a unique large extracellular domain. In sheep, this comprises 341 amino acids (a.a.). This domain is encoded in the *lhr* gene, exons 1–10, which has a signal peptide, a leucine-rich repeat (LRR) region, and is flanked by cysteine-rich regions [2,3].

In addition, LHR binds chorionic gonadotropin (hCG). This is a placental hormone, similar to LH, which is essential for progesterone synthesis during pregnancy. In the testis, LHR is activated by LH, which is also called the interstitial cell-stimulating hormone. This initiates the transduction process via cAMP and also activates the reaction pathways for the synthesizing of testosterone by the Leydig cells [4]. The second large domain of LHR, encoded into the *lhr* gene, comprises the long eleventh exon, which is unique to the GPCR structure and is formed by seven-transmembrane α-helices and a cytoplasmic tail corresponding to the carboxy-terminus [5]. As with most GPCRs, the tertiary structure of LHR is not available.

LHR has been studied in several expressions, for instance, in human embryonic kidney cells [5]. Researchers have studied LHR expression in baculovirus-infected insect cells [6,7]. The parent FSH human receptor has also been expressed in *E. coli* [8]. The authors of [9] identified the X-ray structure of the extracellular domain of the follicle-stimulating hormone receptor (FSHR), complexed with the follicle-stimulating hormone (FSH), which is a glycosylated hormone analogous to LH. These findings have been very useful to researchers comparing the FSHR and LHR receptors [10]. Analysis of the sheep receptor has shown that the whole LHR sequence exhibits 51% similarity, in comparison with FSHR. However, when only the extracellular domains of the two receptors are compared, the sequence similarity declines to 40.5%, not including the initial signal peptide sequence. These data of conserved sequences are of value to researchers; however, in terms of structural biology, they are insufficient for solid molecular modelling purposes; specifically, they do not reveal the extent of the amino acids interacting with LH.

Further studies are, therefore, needed to determine the tertiary LHR structure. Such knowledge may contribute to a better understanding of the hormone receptor interactive mechanism, as well as its physiological implications and possible pharmacological applications. Because our study group has maintained interests in endocrinology and the biology of reproduction, particularly LH in sheep [11], we sought in this study to obtain the extracellular domain of LHR in a heterologous protein expression system, to serve as a foundation for future experiments.

## 2. Materials and Methods

### 2.1. RT-PCR

We extracted total RNA from 250 mg of testicle tissue using the TRIzol (Thermo Fisher Scientific Inc., Carlsbad, CA, USA) method. To obtain Ovis aries cDNA, we treated 1 µg of total RNA with Superscript II reverse transcriptase kit and oligo dT_18_ primer (10 pmol/µL) (Thermo Fisher Scientific Inc.). As stated above, we termed the extracellular domain of LHR as lhr-bed. We then amplified lhr-bed from the synthesized cDNA using the primers 5-CGAATTCCATATGTCACTCACCTACCTCCCTATCAA (forward) and 5-CCGCTCGAGAAGTGTTTCATTATTTGGTCTCCTTGC-3 (reverse), with the restriction sites (underlined) targeted by the enzymes NdeI and XhoI, respectively. The PCR conditions were as follows: 94 °C, 5 min; 94 °C, 1 min; 60 °C, 1 min, and 72 °C, 1 min; allowing for 35 cycles and a final stage of 72 °C for 7 min.

### 2.2. Cloning, Expression and Purification of the rLHR-Bed 

The obtained 762 bp amplicon was cloned into pJET1.2/blunt (Thermo Fisher Scientific Inc.) with CloneJET PCR cloning Kit, subcloned into pCOLD II vector and transformed into BL21 (*DE*3) chemically competent cells (Thermo Fisher Scientific Inc.). The clone that expressed the rLHR-Bed (recombinant extracellular domain luteinizing hormone receptor) fused with a histidine tag, was incubated in a bath at 25 °C for 30 min. Then, to achieve induction, we added IPTG (1 mM) (Merck, Darmstadt, Germany) and carried out incubation in agitation for 18 h at 25 °C, in line with the 25 °C modification protocol suggested in the cold-shock expression system [12].

### 2.3. Purification: Lysis, Column Refolding and Elution

First, the induced pellet (50 mL induction) was lysed with lysozyme (1 µg/mL) (Merck) with 1× complete (Roche, Darmstadt, Germany) in 20 mL of solubilization buffer. This was then incubated in agitation at 4 °C overnight. We then centrifugated at 8000× *g* rpm for 30 min at 4 °C, filtrated the fraction soluble (0.45 µM), and used the pellet to evaluate the insoluble recombinant. Next, we prepared Akta Prime FPLC (GE Healthcare, Freiburg, Germany) for use in manual mode, with ports used as follows: A1: solubilization buffer (20 mM Tris, 0.5 M NaCl, 5 mM imidazole, 8M UREA, and 0.1% CHAPS) (Merck); B: refolding buffer (20 mM Tris, 0.5 M NaCl, 5 mM imidazole, and 0.1% CHAPS) (Merck); A2: elution buffer (20 mM Tris, 0.5 M NaCl, 250 M imidazole, and 0.1% CHAPS) (Merck). We adjusted all buffers to pH 8.0.

**Figure 1:**
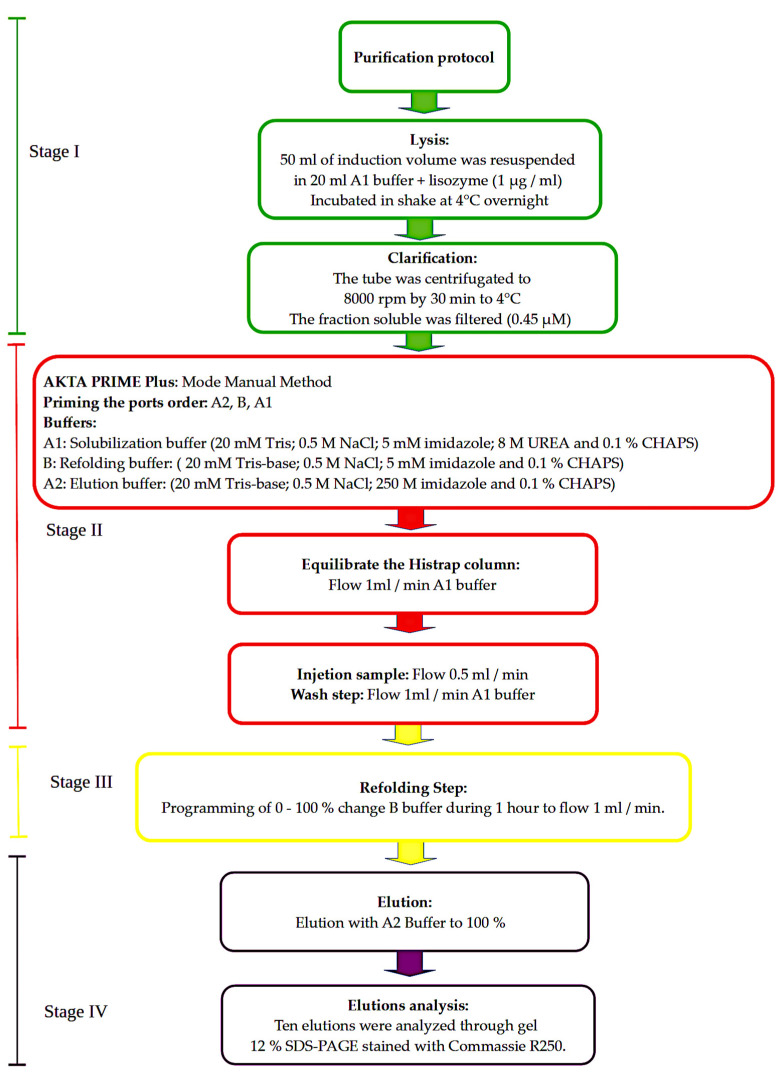
Purification protocol of LHR-Bed. The figure summarizes the four stages of the purification protocol. Stage I (green): lysis and fraction-obtaining. Stage II (red): Akta Prime preparation in manual mode. Stage III (yellow): refolding. Stage IV (purple): elution analysis.

We purified protein using a Ni–NTA HisTrap column (GE Healthcare, Freiburg, Germany). We then primed each of the ports in the following order: A2, B, A. We equilibrated the column with solubilization buffer for 45 min at a flow rate of 1 mL/min. Around 20 mL of sample was charged in the loop, and this was injected at a flow rate of 0.5 mL/min. We carried out the wash step with solubilization buffer at a flow rate of 1 mL/min until stabilization of the line base after approximately 45 min (Figure 1).

After the refolding step, we programmed port B to a 0–100% range with a flow rate of 1 mL/min. Finally, we carried out the elution step with a 100% step of port A2 (elution buffer) (Figure 1). We evaluated the first ten elations by electrophoresis in a 12% SDS-PAGE system stained with Coomassie brilliant blue (Merck).

We confirmed rLHR-Bed identification by means of WB and MS techniques. File S1 shows data of rLHR-Bed detection by anti-His monoclonal antibodies (Merck) and spectrometric masses (MS).

### 2.4. Computational Methods

We carried out computational assessment using the genome assembly Oar_rambouillet_v1.0 of sheep (*Ovis aries)*, along with the NC_040254.1 locus, to reveal the genomic characteristics of *lhr*. We carried out final protein analyses using the Bath-CD-Search function of the Conserved Domain Database V3.20, Bethesda, USA [13], using the TMHMM Server v. 2.0, Lyngby, Denmark [14] and a protein secondary structure prediction server [15]. We used the RNAfold WebServer, Vienna, Austria (rna.tbi.univie.ac.at/cgi-bin/RNAWebSuite/RNAfold.cgi, accessed on 18 August 2022) [16] to predict the secondary structure *cpsA* 5′UTR element in the pCOLD-II vector [17].

## 3. Results

**Figure 2:**
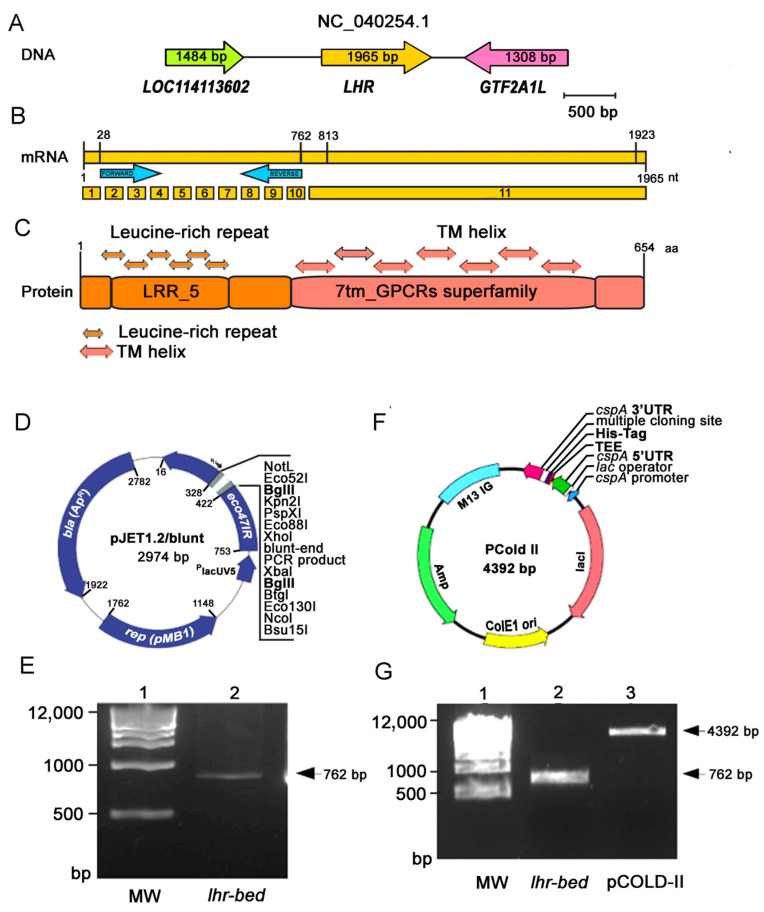
Genomic location of LHR gene in *Ovis aries.* (**A**) shows the LHR gene of 1965 bp length. Upstream and downstream of the LHR genes are the LOC114113602 and GTF2A1L gene loci encoded by 1484 bp and 1308bp, respectively. (**B**) Eleven exons encompass the LHR mRNA. Exons 1 to 10 encode the extracellular domain, while the long exon 11 encodes 7TMD and the carboxy terminal. (**C**) Arrangement of LHR predicted according to NCBI Batch CD-Search, showing 2 domains: a leucine-rich region (LRR) and seven-transmembrane helices characteristic of the GPCR superfamily. (**D**) Cloned *lhr*-*bed* gene. Characteristics of the store plasmid pJET.2/blunt cloning vector for *lhr*-*bed* are shown. (**E**) The amplicon (insert), of 762 bp, corresponding to *lhr-bed* (lane 2). (**F**) Map pCOLD-II expression vector. (**G**) Bands of the expressed vector pCOLD II (lane 3) and the insert of 762 bp yielding the pCOLD II-*lhr-bed* as product expression after digestion by NdeI and XhoI enzymes and ligation with T4 ligase (lane 2). The MW of the DNA marker (1 kb) is shown in B and C, lane 1.

The *Ovis aries lhr* gene was the focus of study in the present work. In sheep, this gene is composed of eleven exons and is located in chromosome three. LH forms a subfamily, along with FSH and TSH receptors, which is characterized by a large ectodomain, and bears a leucine-rich region and glycosylated N-terminus that serves as the binding site of their respective hormones. In this work, by means of a heterologous system, we expressed the recombinant protein of the LHR external domain, here termed *lhr-bed*. We began with the *O. aries* Oar_v4.0 genome assembly with the accession ID GCF_000298735.2 and locus NC_040254.1 of the *lhr* gene, which has a size of 1965 bp (Figure 2A). Related to the *lhr* gene, a downstream genome sequence of 1308 bp is located at the GTF2A1L gene, while an *lhr*-related upstream sequence of 1484 bp is located at the LOC114113602 gene (Figure 2A). The expressed LHR transcription has an mRNA with 1965 bp comprising 11 exons. The extracellular region is encoded by exons 1–10, while the seven-transmembrane domain is encoded by exon 11 (Figure 2B).

Our computational analysis of the large N-terminal extracellular domain, including the LRR, with related LHR–LH binding and the transmembrane region, revealed a complete protein consisting of 654 a.a. (Figure 2C).

However, in the present work, the amplified *lhr-bed* mRNA from *O. aries* testis included only the region between exons 2 to 10, i.e., 762 bp encoding only 273 a.a. (Figure 2B). We, therefore, applied cold-shock expression technology; that is, we cloned and sub-cloned the *lhr-bed* gene ligated to the pCOLD II vector expression. We then sub-cloned the positive-selection cloning vector pJET1-2-blunt-*lhr-bed* for expression and efficient recovery and ligated the DNA insert of 762 bp cloned from pJET1.2/blunt (Figure 2D,E) into the pCOLD II vector. The sub-cloning process included both the NdeI and XhoI digestion enzymes (Figure 2F,G).

We took special care in determining the correct size and alignment of the expression vector and the insert *lhr-bed* gene from agarose gel (Figure 2E–G). Regarding this gel, lane 2 of Figure 2G shows the corresponding insert to *lhr-bed* with 762 bp size, while line 3 of the same figure indicates the linearized pCOLD II, with a size of 4392 bp. Figure 2D–G show the results of cloning and subcloning.

The induction experiment yielded several clones derived from the pCOLD II-*lhr-bed* construction that were transformed into the BL21 strain bacteria. We, therefore, induced the recombinant protein of the extracellular domain of LHR *O. aries* (rLHR-Bed) by experimental means, as follows: we labeled BL21 cells transformed with pCOLD II-empty as negative induction; Clones 2 and 4 were determined as positive, as a result of the transformed pCOLD II-lhr-bed construction.

**Figure 3:**
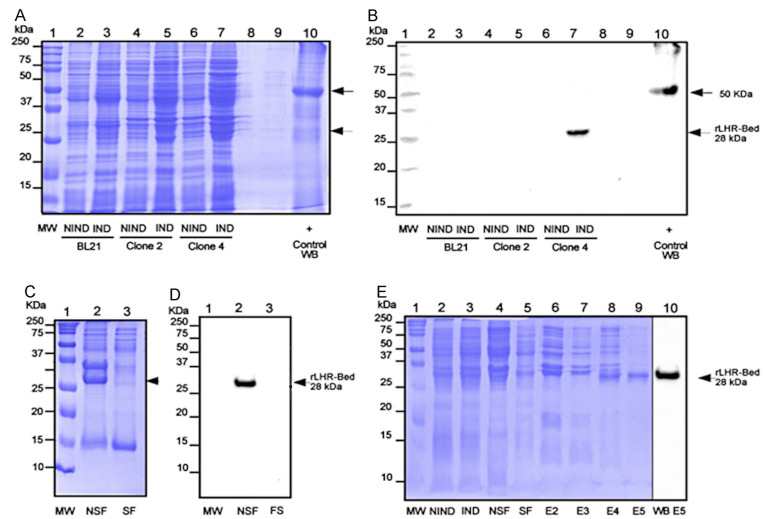
rLHR-Bed induction-solubilization-refolding-purification profile. (**A**) Lane 1 shows MW (molecular weight). Lanes 2–7 show non-induction (NIND) and induction (IND) for BL21 cells without transformation, Clone 2, and Clone 4, respectively. (**B**) Western blot (WB) SDS-PAGE shows rLHR-Bed profiles with detection signals between 25 and 37 kDa; this expression coincides clearly with rLHR-Bed (lane 7). The WB control was a 50 kDa protein with tag histidines (lane 10). Arrows indicate 50 and 28 kDa, respectively. (**C**) Solubility profile test of rLHR-Bed shows: MW (lane 1), non-soluble fraction (NSF) protein, (lane 2), and soluble fraction (SF) protein (lane 3). The arrow indicates rLHR-Bed. (**D**) Western Blot from a gel SDS-PAGE of the rLHR-Bed solubility profile. Lane 1 shows MW; lines 2 and 3 show NSF, and SF, respectively. The presence of rLHR-Bed can be observed in the non-soluble fraction at 28 kDa (lane 2). (**E**) Complete profile of rLHR-Bed purification and its WB detection. Lane 1 shows MW; lanes 2 and 3 show samples of NIND and IND, respectively; lines 4 and 5 show NSF and SF, respectively, lines 6–9 represent elutions 2–5. and 5; lane 10 shows the WB of elution 5.

We evaluated the extent of protein expression using SDS-PAGE profiles. In addition, as WB positive control, we added the recombinant protein with the His-tag of 50 kDa (Figure 3A, lane 10). In the case of BL21 Clone 2, we found no expression of the recombinant protein (Figure 3A, lanes 3, 5). On the contrary, we observed a band of ~28 kDa, corresponding to the induction elicited by Clone 4 (Figure 3A, lane 7). To confirm that the band located between 25 and 37 kDa was actually the rLHR-Bed, we carried out a WB analysis using an anti-His tag antibody mediated with a twin gel corresponding to the protein profile of the induction experiment (Figure 3B).

We also submitted the rLHR-Bed induction elicited by Clone 4 to solubility evaluation. The resulting SDS-PAGE exhibited a marked band in the insoluble fraction, especially in the 25–37 kDa range, while the soluble fraction was less intense within this range (Figure 3C). Our WB experiment clearly confirmed the rLHR-Bed in the insoluble fraction (Figure 3D, lane 2). However, our solubility analyses of rLHR-BED showed that the expressed protein yielded inclusion bodies. This finding compelled us to implement a column protocol, in the form of a denaturation/refolding scheme designed to obtain recombinant protein sufficiently soluble to purify the rLHR-Bed. We achieved the denaturing condition by modifying the solubility buffer to 0.1% CHAPS. This is considered the minimum micellar concentration necessary to improve the solubility of recombinant proteins [18]. Figure 3E shows solubility results, including the complete profile of purification, as revealed by SDS-PAGE (Figure 3E).

Thus, as anticipated, the expressed and purified protein was actually rLHR-Bed, and we obtained this finding by two reliable techniques: WB mediated with anti-histidine monoclonal antibodies and mass spectrometric (MS) analysis. Our final results confirmed that the induced protein did, indeed, correspond to the rLHR-Bed of *O. aries* (Table S2).

Subsequently, we sought to obtain electrophoretic profiles from two vials containing the purified recombinant protein with a concentration of 0.25 mg/ L. The obtained band was cut and sent for MS identification to the proteomic unit of the Instituto de Biotecnología-UNAM, México. Seven peptides were identified, corresponding—with 100% probability—to the lutropin–choriogonadotropin hormone receptor OS = *Ovis aries*, according to the UniProt source database (Table S2). Clearly, the spectrum mass/charge (M/Z) vs. relative intensity ratio corresponding to each peptide showed—positively—the sequenced peptide, protein identification probability, percentage of the best peptide, and spectrum M/Z (Table S2).

**Figure 4:**
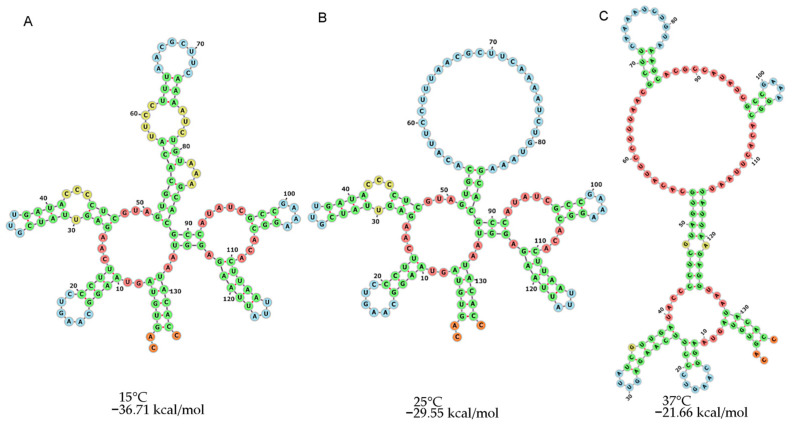
Secondary structure prediction of *cspA* 5′UTR of pCOLDl II under varying temperature conditions. The models were obtained using RNAfold (http://rna.tbi.univie.ac.at/cgi-bin/RNAWebSuite/RNAfold.cgi, accessed on 18 August 2022) and showed predictions with the following temperature parameters: (**A**) 15 °C; (**B**) 25 °C; and (**C**) 37 °C.

By adopting a 25 °C induction temperature, we successfully expressed the recombinant extra-membranal protein of LHR in the pCOLD-II system, which was designed to express at 15 °C [17]. To explain this finding, we used the sequence 5′UTR with a secondary structure at 15 °C, which shows a free energy value for the thermodynamic ensemble of −26.71 kcal/mol (see Figure 4A). We modeled the same sequence at 25 °C and found a free energy value for the thermodynamic ensemble of −29.55 kcal/mol (Figure 4B).

Similarly, at 37 °C, the free energy value for the thermodynamic ensemble was −21.66 kcal/mol (Figure 4C), and this sequence conserved 90% of similar secondary structure, compared with those at 15 °C and 25 °C. However, at 25 °C, we recorded a value of 86 nt, compared with the structure of 50 nt at 15 °C.

Interestingly, the 5′UTR loses its structure at 37 °C. Nevertheless, our results clearly show stability and structure conservation at 25 °C, and this explains the induction of the recombinant protein in the system pCOLD-II at 25°C [12].

## 4. Discussion

Membrane proteins constitute 15 to 39% of the human proteome [19]; among these, the G protein-coupled receptors (GCPRs) are a very important group. Many GCPRs are involved in hormone transduction and modulation. However, the purification of those proteins is a challenging task because of low-quantity expression, problems with solubility, and structure stability issues. Today, rhodopsin is the only GCPR that has been well-characterized [20]. However, many strategies have been developed to purify membrane proteins [21,22], and powerful software applications, such as AlphaFold, have been developed for protein structure prediction [23]. With this in mind, we focused upon LHR in this study, a GCPR associated with endocrinology and, especially, the biology of reproduction. We sought to determine the external domain of LHR expressed in the testicle RNA of *Ovis aries*. This was a feasible objective because previous studies confirmed the expression of the *lhr* gene in male rodent Leydig cells [24], which secrete testosterone and promote spermatogenesis [1,2,3].

Previous studies of the *lhr* gene—which contains 11 exons—have been based on data obtained from bioinformatics tools [3,4]. Researchers have found that the first ten exons encode for the extracellular domain and have deduced major characteristics from sequences of nucleotides, amino acids, and experimental results. Such analyses have highlighted the leucine-rich repeat region where the LH hormone binds, as well as the encoding by the eleventh exon of the seven-transmembrane helices constituting the TM domain [3,4].

Our initial strategy involved obtaining the protein termed rLHR-Bed using the method of Spadiut et al. [12], but with temperature increased from 15 °C to 25 °C and with the addition of a pre-induction course, resulting in an optimum induction of ~0.24 mg/L of recombinant protein. By applying this modified method, we successfully induced the rLHR-Bed and showed that the pCOLD II system is stable and functional at 25 °C.

We improved the integrity and solubility of the protein by means of a lysis buffer containing CHAPS and UREA. Membrane protein solubility is affected by changes of temperature in the presence of detergent. Analyses of the kinetics and thermodynamics of membrane protein folding, carried out by Roman et al. [25], showed that temperature affects the stability of folding by altering the delta G, so that the folding protein is different at minimum and maximum temperatures. In addition, protocols concerning the purification of membrane proteins specify the use of detergents, which affect the folding process, in relation to the micellar concentration of the detergent used [18]. In short, membrane protein solubility is determined by a number of parameters, including the concentrations of phospholipids, the detergent used, and temperature, among others. In this study, we found that CHAPS 0.1% and a temperature of 25 °C served to increase protein solubility. The parameters just described can also be adjusted in the purification of other membrane proteins [18].

Comparing our results with those of other models of LHR expression, we note some differences and identify some shortcomings in our technique. For example, in human embryonic kidney cells, the expressed extracellular domain shows high affinity to hCG, but remains trapped within the cells [5]. Similarly, the LHR expressed in baculovirus-infected insect cells is inactive and remains trapped in aggregate pools [26]. In *E. coli*, the extracellular domain of the parent human follicle-stimulating hormone receptor (hFSH) has been expressed in a chimeric fusion model, in which thioredoxin reductase and glutathione reductase genes in the cloning plasmid vector retained the disulfide bonds of the expressed LHR extracellular domain. This finding confirmed that the truncated receptor may show high affinity to hFSH, similarly to the native receptor, even in the absence of glycans [8]. Nevertheless, this elegant design is still quite elaborate. Complex chimeric expression of the extracellular domain of the LH receptor has been achieved, but by less economical means, for instance, in the CD8 lymphocytes membrane [27].

It is important to recall that new genotypes of *E. coli* BL21 (*DE*3) are available, namely C41 and C43 mutated in *lacI*. These are better able to resist the toxicity caused by membrane protein overexpression [28]. Though we obtained a substantial yield in this study using the BL21 (*DE3*) strain, improved results might be obtained using C41 and/or C43. Such a strategy is functional; it increases the solubility of the protein membrane. In addition, regardless of the fact that the insoluble protein aggregate forms inclusion bodies, the protein is able to conserve its native state secondary structure [25]. The positive effect of low temperature on the production of soluble recombinant proteins in *E. coli* has also been demonstrated [25,29].

We can state some advantages of the improved technique used in this work. For example, the disulfide bonds were retained because neither DTT nor β–mercaptoethanol was used. We also avoided the use of dialysis, which allowed for an easy purification procedure, in which the receptor was left in a harmless buffer, ready for future crystallization. The methods described here may also assist in the future for obtaining membrane proteins and in the determination of the biochemical and structural characterization of proteins. In our study group, we are keen to test the binding of LH isoforms in the expressed extracellular LHR motif. In our previous work with LH isoforms, we have already identified some differences between cAMP and vascular endothelial growth factor production [11]. The membrane proteins so obtained could be used for other applications, such as the generation of specific antibodies, which could be used to study native receptors with more specificity, and better reveal the hormone receptor interaction, with possible beneficial physiological and pharmacological consequences. In the medical field, tools might be developed to study the membrane receptors that are closely associated with the onset of neuro-degenerative disorders, cancer, endocrine disorders, and diseases of reproductive biology.

## 5. Conclusions

In this study, we successfully expressed the extracellular domain of LHR using a reliable molecular biology method requiring relatively little effort. We obtained the protein rLHR-bed expressed by the *lhr-bed* gene obtained from testicular Leydig cells in *O. aries*. We selected BL21 (*DE*3) competent cells and the pCOLD II vector to amplify the gene. Drawing on methods previously reported in the literature, we adopted a strategy of induction-solubilization-refolding and purification. Our procedure was β-mercaptoethanol-free and involved no dialysis, resulting in a reduced processing time. By such means, recombinant protein was expressed in a significant quantity and subsequently purified. Our results suggest that this highly economical method could be applied to express almost any membrane protein.

## Data Availability

The datasets generated and/or analyzed during the current study are available from the corresponding authors.

## Supplementary Materials

The following supporting information can be downloaded at: https://www.mdpi.com/article/10.3390/cimb44110387/s1: File S1: Western blot method (WB), spectrometric mass analysis, and spectrometric mass identification; Table S2: rLHR-Bed mass spectrometric identification.
