# Supplementary material for: Single-Step Protocol for Isolating the Recombinant Extracellular Domain of the Luteinizing Hormone Receptor from the Ovis aries Testis
Source: Curr Issues Mol Biol. 2022 Nov 17;44(11):5718–27. doi: 10.3390/cimb44110387 (PMC9688765; doi:10.3390/cimb44110387)
Supplement: Supplementary file 1 [file cimb-44-00387-s001.zip › cimb-1941993-supplementary.pdf]

Supplementary Materials

Single-Step Protocol for Isolating the Recombinant Extracellular Domain of the Luteinizing Hormone Receptor from the *Ovis aries* Testis

José Luis Villalpando-Aguilar [1], Itzel López-Rosas [2,3,*], Arnulfo Montero-Pardo [4], Elisa Azuara-Liceaga [5], Javier de Jesús Valencia-Méndez [6], Cynthia R. Trejo-Muñoz [7] and Carlos Kubli-Garfias [7,*]

[1] Instituto de Investigaciones en Matemáticas Aplicadas y en Sistemas, Universidad Nacional Autónoma de México, Mérida 97302, México
[2] Consejo Nacional de Ciencia y Tecnología, Ciudad de México 03940, México
[3] Colegio de Postgraduados Campus Campeche, Campeche 24050, México
[4] Facultad de Medicina Veterinaria y Zootecnia, Universidad Autónoma de Sinaloa, San Benito 80260, México
[5] Posgrado en Ciencias Genómicas, Campus del Valle, Universidad Autónoma de la Ciudad de México, Ciudad de México 03100, México
[6] Facultad de Medicina Veterinaria y Zootecnia, Universidad Nacional Autónoma de México, Ciudad de México 04510, México
[7] Instituto de Investigaciones Biomédicas, Universidad Nacional Autónoma de México, Ciudad de México 04510, México

***** Correspondence: itzel.rosas@colpos.mx (I.L.-R.); kubli@unam.mx (C.K.-G.)

Supplementary File S1. rLHR-Bed detection, Spectrometric mass and identification methods.

Western Blot method (WB)

The detection rLHR-Bed was performed follow recommendations Towbin 1979 and used a semi-dry transfer system (Scientific) during 20V/25 min. Then the immunoblotting was carried out with antibody Anti-Hexahistidine with a 1:3000 dilution. Next, the membrane was incubated with an Anti-Rabbit anti-mouse IgA HRP conjugated with to 1:5000 dilution and developed using and Quimioluminicente Luminata Tclassico Western HRP Substrate (Merck) and reveled in Chemidoc Imagen System (BioRad).

Spectrometric mass analysis

The peptides obtained by enzymatic cleavage were desalted with Millipore ZipTips C18 and analyzed in an LTQ Orbitrap Velos mass spectrometer connected to an EASY-nLC II nanoflow pump (Thermo Fisher), including a nanoelectrospray ionization source (ESI) and a Fourier transform mass detector with ESI in positive ionization mode. The whole system was calibrated with the Calmix solution; that is: N-butylamine, caffeine, Met-Arg-Phe-Ala peptide and Ultramark 1621. N-butylamine (73.14 Da), was included to extend the mass calibration aimed at lower m/z values. This type of calibration allows the determination of molecular mass with an accuracy of less than 5 ppm.

Regarding the nanoflow liquid chromatography procedure, two solvents were allowed for 120 minutes with a gradient system of 80%-10%, solvent A (water with 0.1% formic acid) and solvent B (water/acetonitrile with 0.1% formic acid). The implemented capillary column was homemade with ID 0.75 µm and 10 cm long RP-C18. The flow of them and 10 cm long RP-C18. The flow of the LC system was set to 300 nanoliters/minute.

Total ion scanning (Full Scan) was performed on an Orbitrap analyzer with a mass resolving power (RP) of 60,000 (RP = m/FWHM). Collision-induced dissociation (CID) and high-energy collision dissociation (HCD) methods were applied for peptide fragmentation. All spectra were acquired in positive detection mode.

The execution and capture of the fragmentation data were performed depending on the total ion scan according to predetermined charges (only ions with z2+, z3+ and z4+ charges were fragmented) with an isolation width of 2.0 (m/z), normalized collision energy of 35 arbitrary units and Q activation of 0.250. Both the activation time and maximum injection time were 10 millise conds per Microscan. During the automatic data capture, the dynamic ion exclusion was as follows: (i) 200 ion exclusion list, (ii) 30 seconds pre-exclusion time, and (iii) 70 seconds exclusion time.

Spectrometric Mass Identification.

Protein identification was achieved by applying the Proteome Discoverer 1.4 program (Thermo-Fisher Co.), and the spectrometric data were encoded in raw format utilizing the Sequest HT search engine. For identity searches, *Ovis-aries*.fasta and *E.coli.*fasta were used from the UniProt protein database (https://www.UniProt.org/). In addition, an FDR (false discovery rate) minimum of 0.01 and maximum of 0.05 were included, and the inverted database (Decoy database) was used as a tool of the "Percolator" validation program.

Special care was taken to establish the maximal tolerance of the molecular mass difference of the ion precursor when comparing the theoretical versus the experimental values, allowing an established precursor of 20 ppm, while the fragment mass tolerance, the dissociation of fragment frequency, was 0.6 Da. Likewise, some constant modifications were established for the automatic search, such as carbamidomethylation of cysteines and variables such as oxidation of methionine (M) and deamination of both asparagine (N) and glutamine (Q).
To validate MS/MS-based peptide and protein identification, the Scaffold program (version 4.8.7) by Proteome Software was applied.

In this way, identified peptides were accepted only if they showed a probability greater than 99.0% according to the scaffold local FDR algorithm. Similarly, identified proteins were accepted only if they could be established, showing at least 99.0% probability, with an FDR of 1.0% and containing at least 2 identified peptides. Protein probability was assigned by the Protein Prophet algorithm developed by Nesvizhskii et al. (2003). Proteins that contained similar peptides but could not be differentiated based on the MS/MS analysis alone were grouped according to the principle of parsimony.

Table S2. rLHR-Bed Mass Spectrometric Identification. The rLHR-Bed mass spectrometric identification showed seven peptides with 100% identity sequence with LHR of *Ovis aries*. The protein probability identification %, best peptide % and m/z spectra are shown.

Sequence peptide	Protein identification probability	Best peptide	Mass spectrum
GATGPSILDISSTK	100.00%	99.70%	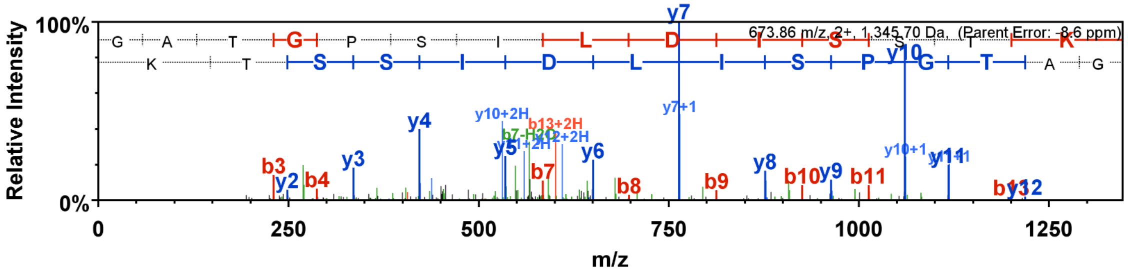
GPSILDISSTK	100.00%	99.70%	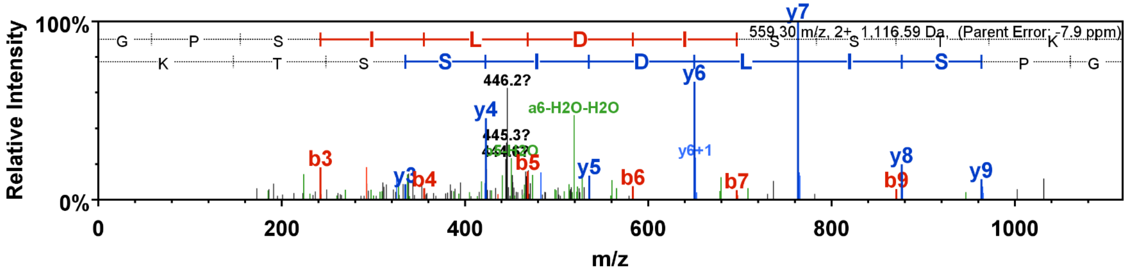
IEISQSDSLEK	100.00%	99.70%	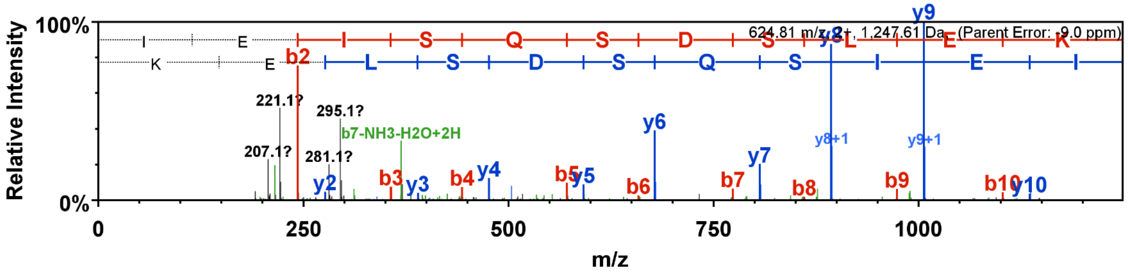

MHNDAFR	100.00%	98.80%	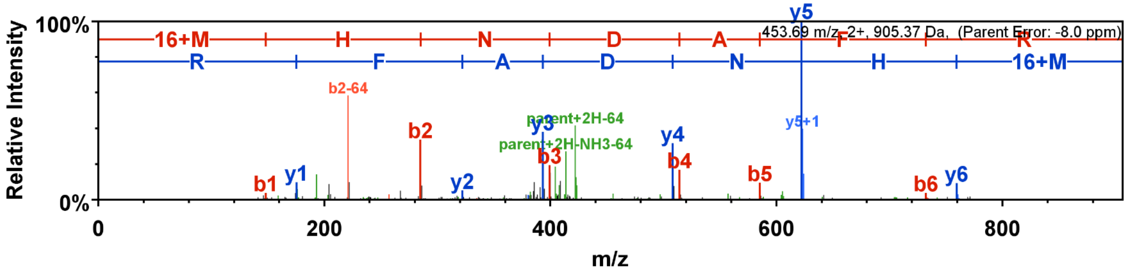
VIPSQAFR	100.00%	99.70%	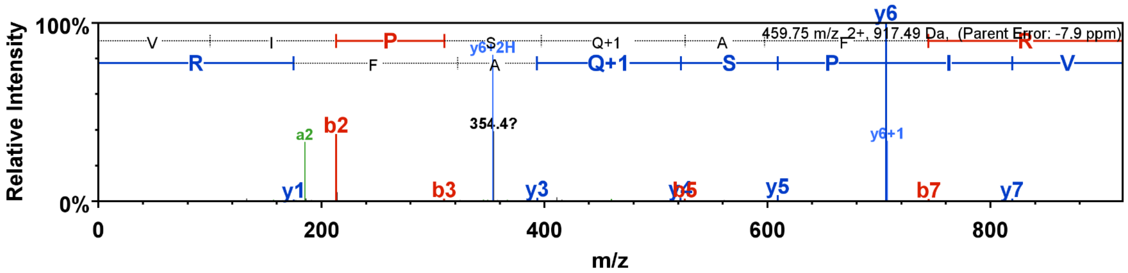
YLSICNTGIR	100.00%	99.70%	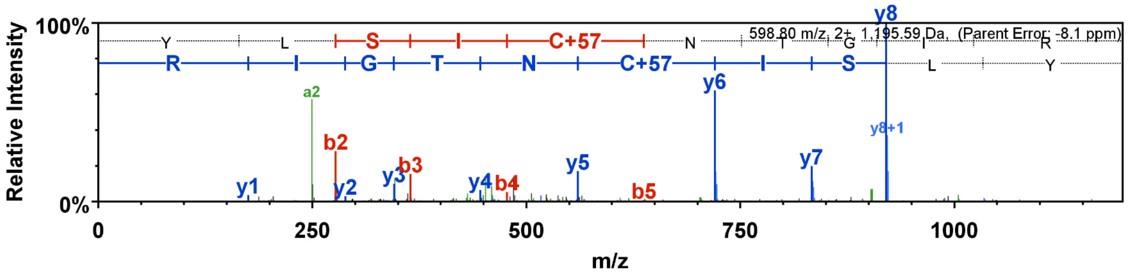
YLSICNTGIRK	100.00%	99.70%	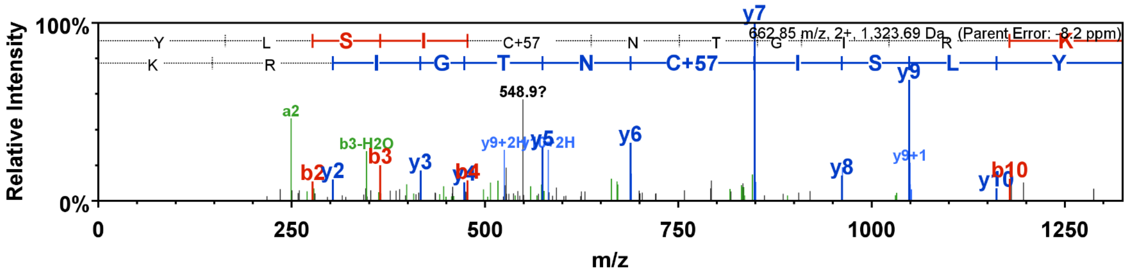
